# Assessing Swedish guidelines for prehospital bleeding control: a cross-sectional appraisal using AGREE II

**DOI:** 10.1186/s13049-026-01720-3

**Published:** 2026-10-30

**Authors:** William Gifting, Linda Johansson, Magnus Andersson Hagiwara, Henrik Hedelin

**Affiliations:** 1 Department of Biomedical and Clinical Sciences (BVK), Linköping University, Linköping, Sweden; 2 Department of Anaesthesiology and Intensive Care, Vrinnevisjukhuset, Norrköping, Sweden; 3 Institute of Clinical Sciences, Sahlgrenska Academy, University of Gothenburg, Gothenburg, Sweden; 4 Faculty of Emergency Medicine, Sahlgrenska University Hospital, Institute of clinical sciences, Sahlgrenska academy, Gothenburg University, Gothenburg, Sweden; 5 PreHospen - Centre for Prehospital Research, Faculty of Caring Science, Work Life and Social Welfare, University of Borås, Borås, SE-501 90 Sweden; 6 Department of Orthopaedics, NU-Hospital Group, Trollhättan, Sweden

**Keywords:** Haemorrhage, Prehospital, Trauma, Guidelines

## Abstract

**Background:**

High-quality clinical practice guidelines are essential for safe and consistent prehospital bleeding control, particularly in settings with variable geography and transport times. This study appraised the methodological quality of Swedish regional prehospital guidelines for traumatic external haemorrhage using the AGREE II instrument.

**Methods:**

This cross-sectional study included clinical practice guidelines requested from all 21 Swedish healthcare regions. Guideline quality was assessed independently by two appraisers using AGREE II, which evaluates 23 items across six domains. Domain scores were calculated as percentages of the maximum possible score.

**Results:**

Complete responses were obtained from 15 of 21 regions. Overall methodological quality was low across the included guidelines. The highest median domain score was observed for clarity of presentation, whereas rigour of development, stakeholder involvement, applicability, and editorial independence scored poorly. Most guidelines lacked explicit descriptions of evidence search strategies, criteria for selecting evidence, links between recommendations and supporting evidence, update procedures, and declarations of editorial independence.

**Conclusions:**

Swedish regional prehospital guidelines for traumatic external bleeding control showed limited methodological quality when assessed using AGREE II. Future guideline development should prioritise transparent evidence linkage, multidisciplinary stakeholder involvement, clear implementation considerations, scheduled updating, and adaptation to regional differences in transport and evacuation times.

**Trial registration:**

Not applicable.

## Background

Trauma is a leading cause of death worldwide, and a substantial proportion of trauma-related fatalities occur before patients reach hospital [1–5]. In both civilian and military settings haemorrhage is an important cause of potentially preventable trauma death [1, 3, 5, 6]. Consequently, effective control of external bleeding in the prehospital environment is critical for reducing mortality [3, 5]. Although adjuncts such as permissive hypotension, early blood transfusion, and tranexamic acid may be valuable, the most immediate and fundamental intervention is timely haemorrhage control [1, 3, 6]. While definitive care is usually surgical, prehospital measures to control bleeding include direct pressure, pressure dressings, wound packing, and tourniquets [7, 8].

Military experience from Western armed forces during the conflicts in Iraq and Afghanistan demonstrated that early tourniquet application could save lives in these setting [9, 10]. This contributed to a marked shift in prehospital guidelines with greater emphasis on early bleeding control in general and tourniquet use in particular. The change was reflected in civilian frameworks such as Prehospital Trauma Life Support (PHTLS) [11], which introduced exsanguinating haemorrhage (X) before airway (A), and in the military Tactical Combat Casualty Care (TCCC) protocol [7], which uses the MARCH algorithm, where M denotes massive haemorrhage. These principles have also been adopted in public health initiatives targeting laypersons, such as the Stop the Bleed campaign [8].

While early and liberal use of prehospital tourniquets has been associated with decreased mortality in high-resource environments with short evacuation times, recent experience from the wars in Ukraine and the Sahel has highlighted significant morbidity and an increased risk of limb loss related to tourniquet use in different operational environments [12–14].

A scoping review identified evacuation time as an important determinant of tourniquet-related morbidity [15]. Civilian prehospital studies have also documented complications with both indication and application technique, including tourniquets applied to bleeding that may have been controlled by other methods and cases where application did not achieve adequate haemorrhage control [14, 16]. Although a non-indicated tourniquet may carry limited risk in settings with short evacuation times, prolonged transport without reassessment can have serious consequences.

Evidence from Iraq, Afghanistan, and subsequent civilian prehospital research has shaped contemporary military and civilian trauma care and has contributed to the development of prehospital guidelines [9, 10]. Clinical practice guidelines are systematically developed statements intended to support evidence-informed healthcare decisions in specified clinical circumstances [17].

Well-constructed CPGs may support consistent, evidence-informed care by summarising evidence in a time-efficient format. Guideline development is resource-intensive and requires reliable processes for identifying and implementing updates. CPGs may also simplify complex clinical situations and therefore need to be developed transparently, with attention to context and applicability. Previous research has demonstrated substantial variation and recurrent methodological and reporting limitations in clinical practice guidelines, including weaknesses in evidence identification, appraisal, recommendation development, applicability, and editorial independence. Such limitations may reduce the trustworthiness and effective implementation of guideline recommendations [17]. In the prehospital field there remains a need for higher-quality studies, clearer methodological reporting, stronger evidence appraisal, and improved implementation of CPGs [18].

The need for context-sensitive guidelines is particularly relevant in Sweden, where geography, transport times, and access to definitive trauma care vary between healthcare regions.

The aim of this study was to appraise the methodological quality of Swedish regional prehospital CPGs concerning traumatic external exsanguination using the Appraisal of Guidelines for Research and Evaluation II (AGREE II) instrument.

## Methods

### Design

This cross-sectional study appraised clinical practice guidelines (CPGs) from all 21 Swedish healthcare regions addressing traumatic external exsanguination, using the Appraisal of Guidelines for Research and Evaluation II (AGREE II) instrument [19].

### Study setting and guideline context

Sweden’s 21 healthcare regions each operate ambulance services coordinated through emergency medical communication centres (EMCCs, dialling 112). Since 2005, Swedish ambulance services have been required to staff all ambulances with at least one registered nurse. Although this represents the national minimum standard, several regions have adopted more stringent staffing requirements and preferentially employ specialist nurses with postgraduate training in ambulance care or prehospital emergency care. Clinical practice guidelines (CPGs) are developed and overseen by regional medical directors. The role of physicians varies between regions and may include Helicopter Emergency Medical Services (HEMS), ground-based critical care units, and remote medical oversight through locally appointed medical directors [20]. A national non-profit organisation, Swedish Collaborative Group of Medical Directors for Ambulance Services (SLAS), brings together regional medical directors and develops national guidelines in line with Advanced Medical Life Support (AMLS) and Prehospital Trauma Life Support (PHTLS) courses [21]. These national guidelines can then be adopted and adapted by individual regions. How regions incorporate these guidelines into their CPG development varies, as they are not required to be used as a baseline when constructing guidelines.

### Data collection and analysis

We requested CPGs from all 21 regions by contacting SLAS and regional medical directors via standardised email, followed by telephone calls providing the same information and invitation, including an AGREE II-based form and supplementary questions [22]. If unsuccessful, we contacted Heads of Operations for regional ambulance services. Data collection was initially planned for three months starting October 2025 but extended to April 2026 due to difficulties establishing contact and low response rates.

Guideline quality was appraised using AGREE II, a validated tool comprising 23 items across six domains (scope and purpose; stakeholder involvement; rigour of development; clarity of presentation; applicability; and editorial independence) (Table 1), each rated on a seven-point Likert scale [23]. AGREE II is a validated instrument for appraising the methodological quality and reporting transparency of clinical practice guidelines [22, 23]AGREE II has been applied in numerous settings globally and recommended by several healthcare organisations for guideline development and appraisal. The appraisers (WG, LJ) completed their initial ratings independently and could not view each other’s ratings until both appraisals had been submitted. They using the MY AGREE PLUS platform [19], with domain scores calculated as percentages of maximum possible score; domain scores were not combined into an overall score. Participating regions were pseudonymised and are reported as Region 1–15 throughout the manuscript; the correspondence between identifiers and regions is not reported. Inter-rater reliability was evaluated using the intraclass correlation coefficient (ICC) based on a two-way random-effects model with absolute agreement and average measures (IBM SPSS Statistics version 28). This model was selected because AGREE II scores were derived from the mean ratings of two independent appraisers. Domain scores were standardised according to AGREE II recommendations, and 95% confidence intervals were estimated using bootstrap resampling.

### Supplementary regional data

In addition to requesting each region’s CPG, we used a structured study form to collect supplementary regional information. The form included questions on the medical director’s specialty, organisation of guideline development, haemorrhage-control education and training, availability of tourniquets, pressure dressings, wound-packing materials and haemostatic dressings, and selected implementation resources. Missing or unclear responses were followed up through telephone contact where possible. These supplementary data were analysed separately from the guideline documents and did not contribute to the AGREE II item, domain, or overall-assessment ratings. Closed-response data were summarised descriptively using counts, and free-text responses were summarised narratively; no formal qualitative analysis was undertaken.

**Table 1:** AGREE II domain description

Domain	Description
Scope and purpose	Overall aim, health questions, target population
Stakeholder involvement	Appropriate stakeholders and views of intended users
Rigor of development	Evidence gathering/synthesis, recommendation formulation and updating
Clarity of presentation	Language, structure and format
Applicability	Barriers/facilitators, uptake strategies and resource implications
Editorial independence	Recommendations not being unduly biased by competing interests; AGREE II items also cover funding-body influence and competing interests

## Results

### Guideline collection

Of the six regions that did not provide a complete response, two declined because the medical director position was vacant, two submitted incomplete material, one declined for another reason, and one agreed to participate but did not submit the requested material.

### AGREE II appraisal

Overall AGREE II scores were low. The median overall score was 8%, with the highest overall assessment score being 42% and the lowest being 0%. No guideline achieved a perfect score in any domain.

Scores varied considerably by domain (Table 2). Domain 4 (Clarity of Presentation) yielded the highest median score (14%; range: 6–39%). Domain 6 (Editorial Independence) received uniformly low scores (median: 0%). Domains 1, 2, 3, and 5 had medians of 3%, 3%, 1%, and 4%, respectively.

Inter-rater reliability of the AGREE II domain scores was moderate to good when based on the mean ratings of two independent appraisers. The pooled domain-level ICC for domains 1–5 was 0.69 (95% CI 0.44–0.83). Domain 1 (Scope and Purpose) demonstrated the highest reliability (ICC = 0.71, 95% CI 0.30–0.92), whereas Domain 5 (Applicability) demonstrated the lowest reliability (ICC = 0.33, 95% CI 0.00–0.73). The corresponding ICC values were 0.47 for Stakeholder Involvement, 0.45 for Rigour of Development, and 0.41 for Clarity of Presentation. Editorial Independence could not be analysed using ICC because all ratings were identical, resulting in zero variance, although absolute agreement between appraisers was 100%.

**Table 2:** AGREE II domain scores across Swedish regional prehospital clinical practice guidelines

Domain	Median (%)	Highest (%)	Lowest (%)
1. Scope and Purpose	3	31	0
2. Stakeholder Involvement	3	19	0
3. Rigour of Development	1	9	0
4. Clarity of Presentation	14	39	6
5. Applicability	4	15	0
6. Editorial Independence	0	0	0

**Table 3:** Summary of AGREE II scores across all CPGs evaluated

Region ID	D1	D2	D3	D4	D5	D6	Overall Assessment
Region 1	11%	19%	9%	31%	15%	0%	42%
Region 2	31%	0%	2%	39%	10%	0%	42%
Region 3	6%	11%	3%	28%	8%	0%	33%
Region 4	8%	0%	2%	22%	10%	0%	33%
Region 5	6%	3%	0%	36%	10%	0%	33%
Region 6	0%	0%	0%	28%	10%	0%	33%
Region 7	3%	11%	1%	8%	4%	0%	17%
Region 8	0%	8%	6%	14%	2%	0%	8%
Region 9	6%	3%	0%	8%	2%	0%	8%
Region 10	3%	0%	0%	14%	0%	0%	8%
Region 11	8%	3%	1%	11%	2%	0%	8%
Region 12	3%	0%	0%	11%	4%	0%	8%
Region 13	3%	0%	0%	17%	2%	0%	0%
Region 14	3%	3%	1%	6%	4%	0%	0%
Region 15	3%	0%	0%	6%	0%	0%	0%

Responses to forms diverged substantially from guideline content: although some regions reported multi-professional working groups in their forms, none of the actual guidelines documented this, resulting in minimum scores for item 4.

All guidelines received the lowest possible score for items 7, 8, and 9 (systematic evidence search, evidence selection criteria, and strength/limitations of evidence), indicating absence of documented scientific methodology (Table 3). References were only included in one guideline, and only one guideline reported a last update date (2018). No guideline described an updating procedure.

### Supplementary questionnaire and follow-up data

The following findings were derived from the supplementary study form and follow-up contacts, not from the CPG documents used for AGREE II scoring.

Among the 15 participating regions, 12 medical directors had specialised in anaesthesia and intensive care; fewer reported emergency medicine or general medicine. Six regions had multiple medical directors and three reported multiple specialities without further specification.

Regarding education, 10 regions reported PHTLS as their primary haemorrhage control training, while 6 reported Stop the Bleed (STB). Twelve regions reported practical and theoretical training under their own authority. One region specified allocated training time (100 h/year/employee).

Availability of haemorrhage-control equipment was generally adequate: equipment sets provided by 14 of 15 regions contained tourniquets and wound-packing materials; those provided by 13 regions contained pressure dressings; and five regions reported haemostatic gauze. Several regions declined to disclose equipment quantities.

Additional questionnaire comments and medical director interviews yielded further insights. Five regions reported equipment quantities. One medical director cited insufficient scientific evidence for haemostatic dressings; two others noted high costs.

Blood product delivery systems for prehospital care were also mentioned as an area of ongoing development in three regions.

One region highlighted rural geographical and demographic challenges, including significant population fluctuations between tourism seasons. The region’s response included trauma care training for staff at community health centres.

## Discussion

This study found low documented methodological quality and reporting transparency in Swedish regional prehospital clinical practice guidelines for traumatic external bleeding control. The main weaknesses concerned rigour of development, stakeholder involvement, applicability, and editorial independence, whereas clarity of presentation received comparatively higher scores. In particular, the guidelines rarely documented systematic evidence searches, criteria for selecting and appraising evidence, explicit links between evidence and recommendations, updating procedures, or competing interests. These findings should primarily be interpreted as limitations in the documented guideline-development process rather than as evidence that the clinical recommendations themselves are inappropriate or that regional ambulance services lack relevant competence, training, or equipment.

The findings are consistent with previous guideline-appraisal research. Studies of prehospital and other clinical practice guidelines have identified recurrent weaknesses in stakeholder involvement, rigour of development, applicability, and editorial independence [17, 24]. The particularly low scores for Rigour of Development in the present study are important because users need to be able to determine whether recommendations are based on systematically identified evidence, expert consensus, adaptation of existing guidance, or local practice. This is especially relevant for prehospital haemorrhage control, where recommendations concerning tourniquet use, wound packing, reassessment, and transport may need to account for patient condition, mechanism of injury, evacuation time, and access to definitive haemorrhage control.

The Swedish prehospital context further illustrates the importance of distinguishing evidence-based principles from operational adaptation. Responsibility for healthcare is decentralised to 21 regions, and geography, transport times, access to HEMS, and proximity to definitive trauma care vary across the country [20, 25–27]. Regional variation in guidelines is therefore not necessarily undesirable. Rather, local adaptations should be transparent and, where possible, explicitly linked to the evidence and operational circumstances that justify them. This may be particularly important in areas with prolonged transport times, where recommendations developed for settings with rapid access to definitive care may not always be directly transferable.

SLAS provides nationally developed prehospital guidance that may serve as a basis for regional guidelines [21]. However, individual regions are not required to adopt these recommendations unchanged, and the extent to which regional CPGs adopt, adapt, or depart from national guidance may vary. The present study did not include the SLAS guideline in the AGREE II appraisal because the predefined unit of analysis was the region-issued CPG. Nevertheless, the relationship between national guidance and regional CPGs is relevant when interpreting the findings. Future research could separately appraise the national guidance and systematically examine how regional guidelines reference, adopt, adapt, or depart from it.

The supplementary regional data provide additional context but should be distinguished from the AGREE II appraisal. Most participating regions reported access to relevant haemorrhage-control equipment, and many reported PHTLS, Stop the Bleed, or locally organised practical and theoretical training. Medical responsibility was also commonly held by physicians specialised in anaesthesia and intensive care. These findings suggest that low AGREE II scores should not be interpreted as indicating an absence of clinical resources or training. Rather, they highlight a discrepancy between reported clinical and organisational resources and the extent to which guideline-development methods were documented in the CPGs themselves. Similarly, some regions reported multidisciplinary involvement in the supplementary study form, although this was not documented in the corresponding guideline documents. Because AGREE II assesses what is reported in or alongside the guideline, undocumented development processes cannot be fully appraised or reproduced.

The findings have practical implications for future guideline development. Regional CPGs would benefit from clearer documentation of how evidence was identified and appraised, how recommendations were formulated, which professional and stakeholder groups participated, how competing interests were managed, and when guidelines should be reviewed and updated. Where recommendations are adapted to regional circumstances, the rationale for such adaptation should also be made explicit. Greater methodological transparency would make it easier for clinicians and guideline developers to distinguish evidence-based recommendations from local adaptations and would facilitate future revision when evidence or organisational conditions change.

Overall, the findings suggest that the principal opportunity for improvement lies not necessarily in increasing the amount of guidance available, but in strengthening the transparency and reproducibility of the processes through which regional prehospital CPGs are developed, adapted, and updated. A more structured approach to guideline development could support regional adaptation while maintaining clear links between recommendations, evidence, and the operational context in which the guidelines are intended to be used.

### Limitations

This study has several limitations. First, complete responses were obtained from 15 of 21 regions, and non-response or incomplete submissions may have influenced the findings. The results should therefore be interpreted as an appraisal of the available documents from participating regions rather than as a definitive assessment of all Swedish regional prehospital bleeding-control CPGs. Second, AGREE II assesses the methodological quality and reporting of guideline development rather than the clinical validity or effectiveness of individual recommendations. Consequently, low scores may partly reflect insufficient documentation of development processes rather than the absence of such processes. Third, supplementary questionnaire and follow-up data indicated that some development activities and multidisciplinary involvement were not documented in the guideline documents themselves. These data were not used to modify the AGREE II ratings.

Fourth, the appraisal was conducted by two appraisers. Inter-rater reliability varied between domains, with several ICC estimates below 0.50 and wide confidence intervals. Because average-measures ICCs were used, the estimates reflect the reliability of the mean ratings of the two appraisers rather than that of an individual appraiser. The limited number of appraisers and restricted variation in some domains may also have affected the precision of the ICC estimates. Finally, the appraisal was restricted to region-issued CPGs and did not include a separate AGREE II assessment of the national SLAS guidance. The present findings therefore cannot determine the methodological quality of the national guidance or the extent to which individual regional CPGs systematically adopted or adapted its recommendations.

## Conclusion

Swedish regional prehospital CPGs for traumatic external bleeding control showed low documented methodological quality and reporting transparency when assessed using AGREE II, particularly regarding rigour of development, stakeholder involvement, applicability, updating procedures, and editorial independence.

Future guideline development should prioritise multidisciplinary development teams, explicit links between recommendations and supporting evidence, transparent documentation of evidence-search and appraisal methods, formal revision schedules, and regionally adapted recommendations for settings with prolonged response or evacuation times.

## Data Availability

The questionnaires and guidelines used and analysed during this study are available from the corresponding author on reasonable request.

## Abbreviations

AGREE II: Appraisal of Guidelines for Research and Evaluation II
AMLS: Advanced Medical Life Support
CPG: Clinical practice guideline
EMCC: Emergency medical communication centre
EMS: Emergency medical services
HEMS: Helicopter Emergency Medical Services
MARCH: Massive haemorrhage, Airway, Respiration, Circulation, Hypothermia/Head injury
PHTLS: Prehospital Trauma Life Support
SLAS: Swedish Collaborative Group of Medical Directors for Ambulance Services; STB: Stop the Bleed
SweTrau: Swedish Trauma Registry
TCCC: Tactical Combat Casualty Care
